# Long-term follow-up of respiratory function in facioscapulohumeral muscular dystrophy

**DOI:** 10.1007/s00415-022-10990-7

**Published:** 2022-02-11

**Authors:** Sjan Teeselink, Sanne C. C. Vincenten, Nicol C. Voermans, Jan T. Groothuis, Jonne Doorduin, Peter J. Wijkstra, Corinne G. C. Horlings, Baziel G. M. van Engelen, Karlien Mul

**Affiliations:** 1 Department of Neurology, Donders Institute for Brain, Cognition and Behaviour, Radboud University Medical Center, Reinier Postlaan 4 (910), PO Box 9101, 6500 HB Nijmegen, The Netherlands; 2 Department of Rehabilitation, Donders Institute for Brain, Cognition and Behaviour, Radboud University Medical Center, Nijmegen, the Netherlands; 3 Department of Pulmonary Diseases/Home Mechanical Ventilation, University of Groningen, University Medical Center Groningen, Groningen, The Netherlands; 4 Groningen Research Institute for Asthma and COPD (GRIAC), University of Groningen, University Medical Center Groningen, Groningen, The Netherlands; 5 Department of Neurology, Medical University Innsbruck, Innsbruck, Austria

**Keywords:** Facioscapulohumeral muscular dystrophy, Respiratory function, Spirometry, Long-term follow-up

## Abstract

**Objective:**

To evaluate the 5-year change in respiratory function in patients with facioscapulohumeral muscular dystrophy (FSHD).

**Methods:**

Genetically confirmed patients with FSHD aged ≥ 18 years were examined twice over five years. Forced vital capacity (FVC) and forced expiratory volume in 1 s (FEV1) were measured using hand-held spirometry with a face mask. Several clinical outcome measures were correlated to respiratory function.

**Results:**

Ninety-two patients were included (57% male, age 18–75 years). At baseline, the spirometry outcomes of 41 patients showed a restrictive ventilatory pattern (FVC < 80% and FEV1/FVC ≥ 70% of predicted) and of 48 patients at follow-up. The mean FVC decreased from baseline to follow-up from 79.0 to 76.7% predicted (*p* = 0.021). This decrease was driven by a subgroup of 15 patients who had a deterioration of FVC of > 10% predicted. The subgroup of 15 patients was more severely affected at baseline (*p* = 0.002 for FSHD clinical score and 0.007 for Ricci score). They developed more frequently spinal and thorax deformities (*p* < 0.001 for kyphoscoliosis and 0.012 for pectus excavatum) and had a larger decline in axial muscle function (*p* = 0.020). Only weak correlations were found between the change in FVC% predicted and the change in clinical scores between baseline and follow-up**.**

**Interpretation:**

Respiratory function remained stable in most patients with FSHD, but a subgroup of patients showed a pronounced deterioration. They showed more severe muscle weakness including the leg muscles at baseline (Ricci score ≥ 6), had spinal and thorax deformities and a relatively fast decline in axial muscle function at follow-up.

## Introduction

Facioscapulohumeral muscular dystrophy (FSHD) is a slowly progressive, inherited muscle disorder and is one of the most prevalent muscular dystrophies [1]. The muscles of the face and shoulder girdle are asymmetrically affected, followed by trunk, pelvic girdle and lower limb muscles [2].

A less prominent feature of FSHD is respiratory function impairment. Approximately 1% of the FSHD population has respiratory insufficiency requiring chronic non-invasive ventilation [3]. However, the proportion of patients with FSHD that have a mild restrictive ventilatory pattern is larger and ranges from 10 to 39% [4–7]. In FSHD, a restrictive ventilatory pattern is caused by weakness of the expiratory abdominal muscles [8], sometimes of the diaphragm, and by chest wall deformities [3, 5, 9, 10].

Currently, the evidence-based guideline on FSHD advises to examine baseline respiratory function in all patients with FSHD and monitor patients regularly if they show an impaired baseline respiratory function or in case of any combination of severe proximal weakness, wheelchair dependence, (kypho-)scoliosis or comorbid conditions that may affect ventilation [7]. Longitudinal data on the decrease of respiratory function in FSHD, necessary to optimize patient management, are scarce. One study described a mean decline of FVC of 3.6% predicted per year in ten patients [5]. Considering the retrospective design and that all patients were assigned to the ‘severe respiratory involvement group’, defined as patients with FVC < 50%, this study does not provide sufficient information about the decrease of respiratory function in FSHD in general. To expand this knowledge, prospective longitudinal data in a larger and more diverse cohort of FSHD patients are needed.

This study aims to assess respiratory function in FSHD over long-term follow-up in a large cohort of patients with FSHD and to identify patients who are more prone to rapid deterioration of respiratory function.

## Methods

### FSHD-FOCUS study

The baseline data of genetically confirmed FSHD patients aged 18 years and older were collected in a large natural history study on FSHD (FSHD-FOCUS study) at the Neurology department of the Radboud University Medical Center, Nijmegen, the Netherlands in 2014 and 2015 [11]. Patients who were able to visit the outpatient clinic to undergo respiratory function testing were included in this longitudinal study. Approximately five years after the baseline visit patients were invited for a follow-up visit. Demographic characteristics of all patients were registered. Patients were inquired about pulmonary comorbidities and use of chronic non-invasive ventilation.

### Respiratory function testing

Respiratory function was examined by hand-held spirometry (Carefusion Microloop Spirometer, Rochester, England) as previously reported [12]. The forced vital capacity (FVC) and forced expiratory volume in 1 s (FEV_1_) were measured in liters. Spirometry was performed in a seated position. Patients were instructed to exhale and then take a maximum inhalation and exhale with maximum speed and effort. For each trial, a facemask was used. After three attempts, the best score was recorded. Results were calculated to percentage of predicted, based on age, sex and height, using the prediction equations for facemask spirometry [12]. A restrictive ventilatory pattern was defined as FVC < 80% predicted and a FEV_1_/FVC ratio of ≥ 70% predicted [3, 5].

### Clinical outcome measures

Manual muscle testing (MMT) was performed using the Medical Research Council (MRC) scores for the following muscles: neck flexors and extensors, shoulder external rotators, shoulder adductors and abductors, elbow flexors and extensors, wrist flexors and extensors, hip flexors, hip abductors, knee flexors and extensors, foot dorsiflexors and plantar flexors. All individual MMT scores were added up to calculate a MMT sum score, ranging from 0 to 140, in which lower scores indicate more severe muscle weakness.

To assess the subjects’ functional abilities, the Motor Function Measure (MFM) was performed. This scale consists of 32 items organized in three dimensions: standing position and transfers, axial and limb proximal motor function and limb distal motor function [13]. In each subject, the scored points were calculated as percentage of the total amount of points that could be achieved. Additionally a ‘MFM trunk’ score was calculated by adding up the scores of items 1, 2, 7, 8, 10, 13, 14 and 25 of the MFM and was displayed as percentage of the maximum amount of points.

The Ricci score was determined to assess clinical severity [14]. This scale ranges from 0 to 10, in which 0 indicates no muscle weakness and 10 wheelchair dependency. Additionally the FSHD clinical score was recorded, which is a 15-point sum score that consists of six independent scores of separately evaluated muscle regions: the face, scapular girdle, upper limb, pelvic girdle, lower limb and abdominal muscles [15]. The presence of spinal deformities, consisting of lumbar hyperlordosis and kyphoscoliosis, and pectus excavatum were examined in each patient. Baseline and follow-up measurements were performed by two different clinicians (K.M. and S.V.).

### Statistical analysis

Data analysis was carried out using IBM SPSS Statistics 25. Population characteristics and demographics were presented using descriptive statistics, including mean and standard deviation and median and interquartile range (IQR). To analyze the differences in respiratory function measures and clinical outcome measures between baseline and follow-up, a paired-samples t test was used for normally distributed continuous variables, a Wilcoxon signed-rank test for non-normally distributed continuous variables and a McNemar’s test for dichotomous variables. Spearman's rho correlation coefficients (CC) were calculated to demonstrate correlations. To analyze the difference in clinical outcome measures between two groups of patients, a Mann–Whitney *U* test was used for non-normally distributed continuous data and a Fisher’s Exact test for dichotomous data. Statistical significance was defined as *p* < 0.05.

## Results

### Patients

At baseline, 164 patients underwent spirometry. Seventy-two patients were lost to follow-up (deceased *n* = 4; unwilling to participate *n* = 27; no spirometry performed during follow-up visit due to COVID-19 regulations *n* = 26, unable to visit the Radboud University Medical Center *n* = 15) and subsequently 92 patients (57% male) were included in this study. The mean age at baseline was 48.4 years (SD 15.3, range 18–75) and disease duration 21.3 years (SD 16.8, range 0–59). Eighty-eight of the 92 patients (95.7%) had FSHD type 1, with a median D4Z4 repeat array size of 6 units (range 2–9). Four patients had FSHD type 2 based on the combination of hypomethylation of D4Z4 repeat array on chromosome 4qA and an *SMCHD1* pathogenic variant. Further patient characteristics and clinical outcome measures are listed in Table 1. Mean time until follow-up was 55.9 months (SD 3.0, range 49–63). Of the 92 patients included in this study, one had chronic obstructive pulmonary disease (COPD) and five were previously diagnosed with asthma. One patient used chronic non-invasive ventilation during baseline and follow-up and one patient started using continuous positive airway pressure (CPAP) at night between the baseline and follow-up visit.

**Table 1:** Respiratory function outcomes and clinical outcome measures at baseline and follow-up

	Respiratory decline (*n* = 15)		No respiratory decline (*n* = 77)	
	Baseline	Follow-up	Baseline	Follow-up
FVC, mean % predicted (SD)	79.0 (19.7)^a^	61.7 (19.4)**	79.0 (12.0) ^a^	79.6 (13.8)
FEV_1_, mean % predicted (SD)	70.8 (16.6) ^a^	58.9 (20.3)**	69.9 (14.1) ^a^	72.9 (13.8)**
FEV_1_/FVC, mean % predicted (SD)	88.1 (8.1) ^a^	92.3 (9.8)	86.0 (11.0) ^a^	89.2 (9.1)*
D4Z4 repeat array size				
2—3, *n* (%) 4—6, *n* (%) 7—9, *n* (%)	1 (6.7) 7 (46.6) 7 (46.6)		6 (7.8) 35 (45.5) 32 (41.6)	
Ricci score, median (IQR)	7.0 (6.0–8.0)	8.0 (6.0–9.0)	6.0 (3.0–7.0)	6.0 (3.0–8.0)**
FSHD clinical score, median (IQR)	9.0 (7.0–12.0)	11.0 (6.0–13.0)	6.0 (3.0–9.0)	7.0 (3.0–10.0)**
MMT sum score, median (IQR)	106.0 (100.0–120.0)	102.0 (94.0–122.0)	127.0 (111.5–135.0)	125.0 (110.0–135.0)*
MFM trunk score % of total, median (IQR)	87.5 (83.3–91.7)	79.2 (62.5–91.7)**	95.8 (88.5–100.0)	95.8 (80.2–100.0)**
MFM score % of total, median (IQR)	79.2 (62.0–87.0)	62.5 (53.7–84.9)**	94.3 (75.8–99.5)	90.1 (65.9–98.2)**
Spinal deformities				
Lumbar hyperlordosis, *n* (%) Kyphoscoliosis, *n* (%)	3 (20.0) 0 (0)	3 (20.0) 6 (40.0)*	5 (6.5) 2 (2.6)	12 (15.6) 3 (3.9)
Pectus excavatum, *n* (%)	2 (13.3)	4 (26.7)	3 (3.9)	3 (3.9)
Use of wheelchair, *n* (%)	0 (0)	0 (0)	2 (2.6)	5 (6.5)

*FVC* forced vital capacity, *FEV*_*1*_ forced expiratory volume in 1 s, *IQR* interquartile range, *MFM* motor function measure, *MMT* manual muscle testing

*p value < 0.05 of the comparison between baseline and follow-up

**p value < 0.01 of the comparison between baseline and follow-up

^a^*p* values of the comparison of baseline spirometry results between the groups were 0.997 for FVC, 0.834 for FEV_1_ and 0.494 for FEV_1_/FVC

### Baseline respiratory function testing

Baseline respiratory function measures are displayed in Table 1. In 41 patients (44.6%), a restrictive ventilatory pattern was present. This group consisted of 40 FSHD1 patients and one FSHD2 patient.

### Longitudinal respiratory function testing

**Fig. 1:**
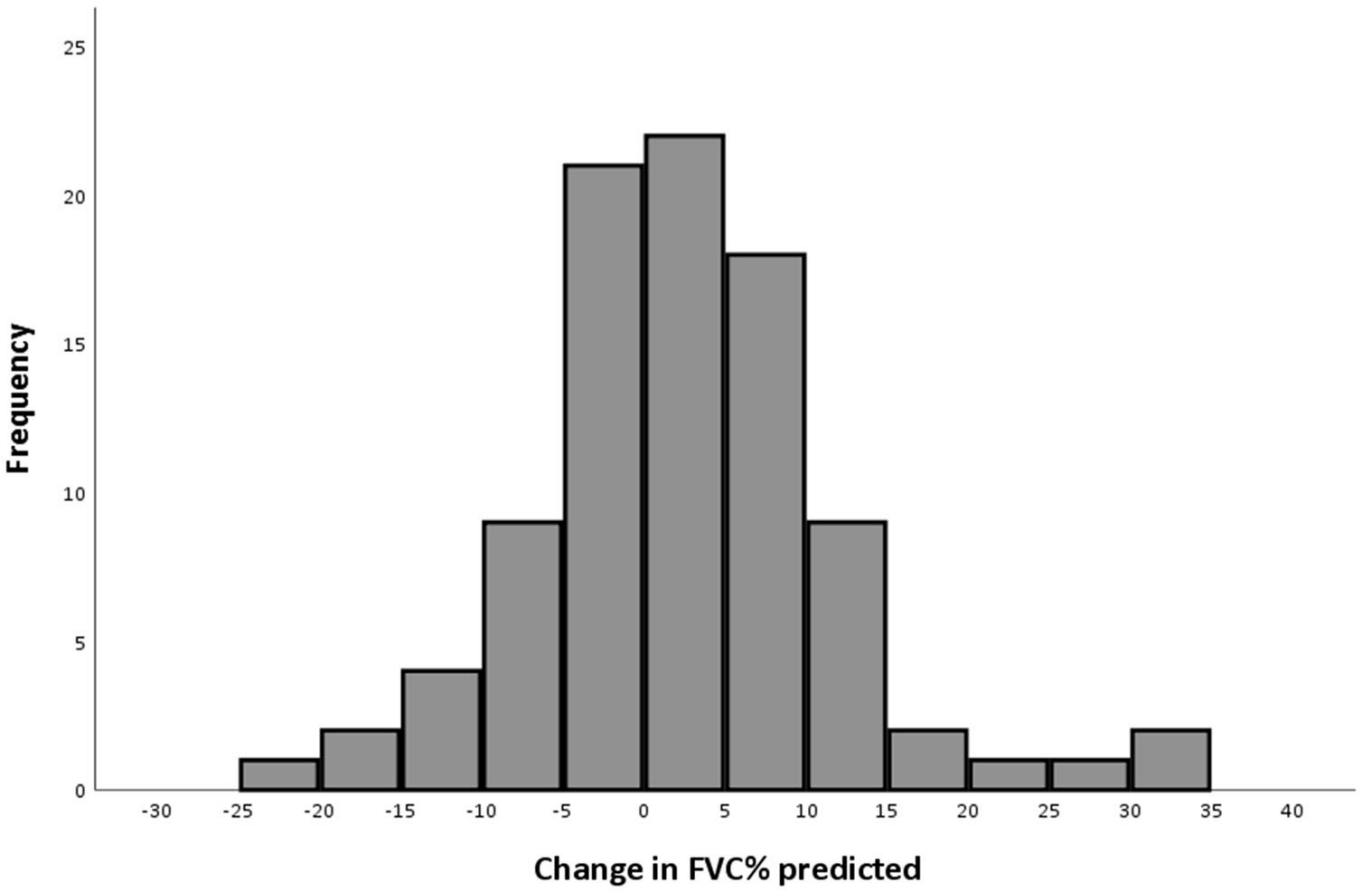
Histogram of change in FVC% of predicted between baseline and follow-up. Mean change in FVC% predicted was 2.3 (SD 9.4). Change values were calculated by subtracting the follow-up value from the baseline value

Overall mean FVC decreased from 79.0% (SD 13.4) to 76.7% predicted (SD 16.2) (*p* = 0.021) and mean FEV_1_/FVC increased from 86.3% (SD 10.6) to 89.7% predicted (SD 9.2) (*p* = 0.003) in five years. However, a large variance was seen in change of respiratory function over time (Fig. 1). The change in FVC% predicted between baseline and follow-up ranged from 33.5% decrease to 23.6% increase from baseline. The same is observed in FEV_1_/FVC% predicted: ranging from 29.0% decrease to 40.6% increase from baseline. This range of change in FVC% predicted in severely affected patients (Ricci score ≥ 7 at baseline, *n* = 35) was − 19.3 to 33.5 and was − 23.6 to 30.5 in moderately affected patients (Ricci score < 7 at baseline, *n* = 57). At follow-up, 48 patients (52.2%) showed a restrictive ventilatory pattern.

Fifteen patients had a deterioration of FVC% predicted of more than 10%, of which four had a deterioration of FVC% predicted of more than 20%. These patients were all FSHD type 1 patients and none used a wheelchair at baseline or follow-up. None of these patients had pulmonary comorbidities or used chronic non-invasive ventilation prior to this study. The patient who started using CPAP had a deterioration of FVC% predicted of more than 20%. At baseline, the respiratory function of the patients in this subgroup did not differ from the other patients (Table 1). The mean age of this subgroup was significantly higher than the age of the other patients (56.3 (SD 13.2) years vs. 46.8 (SD 15.2) years, *p* = 0.026). The disease duration in this subgroup was also significantly longer than in the other patients (33.7 (SD 16.9) years vs. 18.9 (SD 15.8) years, *p* = 0,001). The proportion of male patients in the two groups did not differ (9 (60%) vs. 43 (55.8%), *p* = 0.786). They did have a significantly higher median Ricci score and FSHD clinical score and a significantly lower MMT sum score, MFM trunk and total score at baseline than the other patients. They more frequently developed kyphoscoliosis and pectus excavatum during follow-up and had a significantly larger decline in MFM score and MFM trunk score between baseline and follow-up (Table 2). The D4Z4 repeat array size did not differ between groups (*p* = 0.694).

**Table 2:** Clinical outcome measures in subgroup of patients with respiratory decline versus patients without respiratory decline

	Respiratory decline (*n* = 15)	No respiratory decline (*n* = 77)	*p* value
BMI at baseline, median (IQR)	24.5 (21.6–28.4)	24.9 (22.1–28.0)	0.994
Ricci score at baseline, median (IQR)	7.0 (6.0–8.0)	6.0 (3.0–7.0)	0.007*
FSHD clinical score at baseline, median (IQR)	9.0 (7.0–12.0)	6.0 (3.0–9.0)	0.002*
MMT sum score at baseline, median (IQR)	106.0 (100.0–120.0)	127.0 (115.5–135.0)	0.002*
MFM trunk score % of total at baseline, median (IQR)	87.5 (83.3–91.7)	95.8 (88.5–100.0)	0.008*
MFM score % of total at baseline, median (IQR)	79.2 (62.0–87.0)	94.3 (75.8–99.5)	0.012*
BMI at follow-up, median (IQR)	26.8 (22.3–30.4)	24.9 (23.0–28.1)	0.305
Spinal deformities at follow-up			
Lumbar hyperlordosis, *n* (%) Kyphoscoliosis, *n* (%)	3 (20.0) 6 (40.0)	12 (15.6) 3 (3.9)	0.706 < 0.001*
Pectus excavatum at follow-up, *n* (%)	4 (26.7)	3 (3.9)	0.012*
Change in Ricci score, median (IQR)	− 1.0 (− 1.0–0)	− 1.0 (− 1.0–0)	0.878
Change in FSHD clinical score, median (IQR)	0 (− 1.0–0)	− 1.0 (− 2.0–0)	0.323
Change in MMT sum score, median (IQR)	4.0 (-4.0–10.0)	1.0 (− 1.0–5.0)	0.348
Change in MFM trunk score % of total, median (IQR)	8.3 (0.0–25.0)	0.0 (0.0–6.3)	0.020*
Change in MFM score % of total, median (IQR)	13.0 (1.1–17.7)	2.6 (0.0–8.1)	0.021*

Change values were calculated by subtracting the follow-up value from the baseline value

*BMI* Body Mass Index, *FVC* forced vital capacity, *IQR* interquartile range, *MFM* motor function measure, *MMT* manual muscle testing

### Correlation between FVC and clinical outcome measures

Correlations between FVC and clinical outcome measures are listed in Table 3. At baseline, the FVC% predicted correlated weakly with all clinical outcome measures. Change in FVC% predicted from baseline to follow-up did not correlate to clinical outcome measures at baseline. Change in FVC% predicted from baseline to follow-up only correlated weakly to change in MFM (CC 0.245, *p* = 0.019) and not to change in other clinical outcome measures used.

**Table 3:** Correlations between FVC% predicted and outcome measures

FVC% predicted at baseline vs.	Spearman's rho CC	*p* value
Age at baseline	− 0.035	0.740
BMI at baseline	− 0.014	0.898
Ricci score at baseline	− 0.297	0.004*
FSHD clinical score at baseline	− 0.311	0.003*
MMT sum score at baseline	0.268	0.010*
MFM trunk score % of total at baseline	0.373	< 0.001*
MFM score % of total at baseline	0.360	< 0.001*

Change in FVC% predicted vs.	Spearman's rho CC	*p* value
Age at baseline	0.187	0.075
BMI at baseline	0.080	0.463
Ricci score at baseline	0.151	0.152
FSHD clinical score at baseline	0.185	0.078
MMT sum score at baseline	− 0.204	0.051
MFM trunk score % of total at baseline	− 0.184	0.080
MFM score % of total at baseline	− 0.187	0.074

Change in FVC% predicted vs.	Spearman's rho CC	*p* value
Change in Ricci score	0.018	0.862
Change in FSHD clinical score	− 0.059	0.577
Change in MMT sum score	0.087	0.412
Change in MFM trunk score % of total	0.205	0.050
Change in MFM score % of total	0.245	0.019*

Change values were calculated by subtracting the follow-up value from the baseline value

*BMI* Body Mass Index, *CC* correlation coefficient, *FVC* forced vital capacity, *MFM* motor function measure, *MMT* manual muscle testing

## Discussion

In this study, we longitudinally examined the respiratory function of 92 patients with FSHD. We found that 45% of the patients already had a restrictive ventilatory pattern at baseline. This proportion varied widely in previously conducted studies. One retrospective study in a similar cohort using the same criteria reported that 38.3% of their cohort had a restrictive ventilatory pattern [5]. A study on respiratory function in patients with suspected respiratory weakness showed more patients with reduced respiratory function (48% with a vital capacity of < 50% predicted) [16]. Other studies showed lower prevalence of a restrictive ventilatory pattern, which could be explained by patients having a lower clinical severity [4], a shorter disease duration [6] or the inclusion of a small homogeneous cohort that did not include severely affected patients [17]. In the study of Wohlgemuth et al., the FVC was within normal range in 63 ambulant patients [9]. However, in that study, ambulant patients were defined as patients who were able to walk using no walking aids, except for ankle–foot orthoses, whereas in this study, patients not using a wheelchair were defined as with a Ricci score of 9 or lower. This variety in proportions has made it difficult to reliably determine the prevalence of respiratory function impairment in FSHD, as mentioned in the evidence-based guideline on the evaluation, diagnosis and management of FSHD by the American Academy of Neurology (AAN) [7]. Our longitudinal study in a large unselected cohort adds important information to help clarify the frequency and severity of respiratory compromise in FSHD.

The results of a previous study suggested a trend toward a higher prevalence of a restrictive pattern in FSHD2 than in FSHD1 patients [4]. However, both in the study of Scully et al. and in our study, the number of FSHD2 patients was small, 8 and 4, respectively. Larger studies are required to determine whether there are differences in respiratory function impairment between FSHD1 and FSHD2 patients. Although this is not within expectation, since the clinical presentation of FSHD2 is similar to FSHD1 [18].

As facial weakness is a key symptom of FSHD [2] using only a mouthpiece instead of a face mask could result in air leakage in the patients with weakness of their oral muscles, especially in forced maneuvers, such as FVC and FEV_1_. Only in the study of Moreira et al., facemasks were used. The other studies did not use them or did not specify. This could have resulted in lower FVC and FEV_1_ values and subsequently an overestimation of the frequency of respiratory function impairment in FSHD [12].

Our study showed a large variance in the change of respiratory function over time, where some patients also showed an increase in respiratory function. This could be caused by (aerobic) exercising, weight loss, respiratory physiotherapy techniques [19] or better patient cooperation. Unfortunately, we did not register these factors. The results could also be influenced by the fact that the baseline and follow-up visits were conducted by two different clinicians.

We identified a subgroup of 15 patients with an evident decline in FVC, whereas the FVC of the other 77 patients remained unchanged. There was no difference in D4Z4 repeat size and BMI between both groups and these characteristics can therefore not be used to determine which patients’ respiratory function will decline over time. The spirometry outcomes at baseline could likewise not be used to distinguish between these patients, since these outcomes did not differ between the groups. The current guideline advises to monitor patients regularly if they have abnormal baseline pulmonary function test results [7]. The results of our study complement this advice and show that baseline pulmonary function is not necessarily a risk factor for respiratory decline. In contrast, the subgroup of patients with a large decline in FVC did have more severe muscle weakness, more functional impairment and higher clinical severity scores at baseline compared to the rest of the cohort. Most of these 15 patients had an FSHD clinical score of 6 or higher and a Ricci score of 6 or higher at baseline, indicating the presence of pelvic or proximal leg weakness. This supports earlier findings that more severely affected patients are more likely to have restrictive respiratory function impairment [4, 6, 8, 9]. We found that kyphoscoliosis and pectus excavatum at five years were more prevalent at follow-up in these 15 patients, which is in line with the previous reports of spinal deformities as a risk factor for respiratory function impairment in FSHD [3, 5, 9]. However, these results could be influenced by the different evaluators at both timepoints. None of the patients in this subgroup used a wheelchair, which is associated with a restrictive ventilatory pattern [4, 5, 9]. The proportion of patients using a wheelchair in this cohort was low, probably because these patients were more frequently unable to visit the outpatient clinic.

Furthermore, this subgroup showed a greater decline in functional scores (MFM trunk and MFM total scores), but remarkably not in muscle strength scores of the upper and lower extremity muscles, compared to the total cohort. An explanation for these findings is that the MFM includes assessment of axial muscles, whereas the MMT sum score and the clinical severity score do not. This leads to the hypothesis that patients with more severe muscle weakness at baseline are more likely to develop spinal deformities due to more severe axial muscle weakness [10], measured as a greater decline in functional scores. This hypothesis combined with the assumption that abdominal muscles and the diaphragm play a direct role in respiratory muscle weakness in FSHD [8], results in a specific group of patients who are prone to develop a more rapid deterioration of respiratory function. With the results of this study, rapid decline in axial muscle function could be added to the factors listed in the current guideline, determining which patients should be monitored regularly for respiratory function deterioration [7].

This study has some limitations. First, we examined respiratory function only by hand-held spirometry. Spirometry is a widely used method of respiratory function testing, but remains an indirect method to evaluate respiratory muscle function and is dependent on patient cooperation. Second, measurement of peak cough flow, maximal inspiratory and expiratory pressures and diaphragm and abdominal muscle ultrasound could have been insightful to extensively evaluate the change in respiratory muscle function. Also, recording of (nocturnal) respiratory complaints would have expanded the characterization of this cohort. Third, we did not assess the FVC in sitting and also supine position which could have added information on diaphragm function as a decline of the FVC when moving from a sitting position to supine position is very suggestive for diaphragmatic weakness [20, 21]. Fourth, as stated before, the measurements at baseline and follow-up were performed by two different evaluators, which could have influenced both respiratory function and clinical outcomes, since both are dependent on patient cooperation and stimulation. To control for variability in spirometry procedures, the second evaluator was trained by the first evaluator. Fifth, expanding the amount of timepoints could have helped in determining the rate of deterioration over time. Lastly, as mentioned above, this cohort could be influenced by selection bias. The most severely affected patients, that frequently use a wheelchair, were often not able to visit the hospital.

In conclusion, this study shows that respiratory function in most patients with FSHD remains stable over a prolonged period of time, but that a subgroup of patients is at risk of deterioration. This subgroup can be identified by severe muscle weakness with involvement of the leg muscles (often a Ricci Score of 6 or higher at baseline), spinal and thorax deformities and a relatively fast decline in axial muscle function. These findings support and enhance the current AAN practice guideline and therefore it should be recommended to take these factors into account when determining which patients should be monitored frequently in clinical practice.

## Data Availability

Anonymized data will be available from the corresponding author upon appropriate request.

## References

[1] Deenen JC, Arnts H, van der Maarel SM (2014). Population-based incidence and prevalence of facioscapulohumeral dystrophy. Neurology.

[2] Mul K, Lassche S, Voermans NC, Padberg GW, Horlings CG, van Engelen BG (2016). What's in a name? The clinical features of facioscapulohumeral muscular dystrophy. Pract Neurol.

[3] Wohlgemuth M, van der Kooi EL, van Kesteren RG, van der Maarel SM, Padberg GW (2004). Ventilatory support in facioscapulohumeral muscular dystrophy. Neurology.

[4] Scully MA, Eichinger KJ, Donlin-Smith CM, Tawil R, Statland JM (2014). Restrictive lung involvement in facioscapulohumeral muscular dystrophy. Muscle Nerve.

[5] Moreira S, Wood L, Smith D (2017). Respiratory involvement in ambulant and non-ambulant patients with facioscapulohumeral muscular dystrophy. J Neurol.

[6] D'Angelo MG, Romei M, Lo Mauro A (2011). Respiratory pattern in an adult population of dystrophic patients. J Neurol Sci.

[7] Tawil R, Kissel JT, Heatwole C (2015). Evidence-based guideline summary: evaluation, diagnosis, and management of facioscapulohumeral muscular dystrophy: Report of the Guideline Development, Dissemination, and Implementation Subcommittee of the American Academy of Neurology and the Practice Issues Review Panel of the American Association of Neuromuscular & Electrodiagnostic Medicine. Neurology.

[8] Henke C, Spiesshoefer J, Kabitz HJ (2019). Respiratory muscle weakness in facioscapulohumeral muscular dystrophy. Muscle Nerve.

[9] Wohlgemuth M, Horlings CGC, van der Kooi EL (2017). Respiratory function in facioscapulohumeral muscular dystrophy 1. Neuromuscul Disord.

[10] Perrin C, Unterborn JN, D'Ambrosio C, Hill NS (2004). Pulmonary complications of chronic neuromuscular diseases and their management. Muscle Nerve.

[11] Mul K, Voermans NC, Lemmers R (2018). Phenotype-genotype relations in facioscapulohumeral muscular dystrophy type 1. Clin Genet.

[12] Wohlgemuth M, van der Kooi EL, Hendriks JC, Padberg GW, Folgering HT (2003). Face mask spirometry and respiratory pressures in normal subjects. Eur Respir J.

[13] Bérard C, Payan C, Hodgkinson I, Fermanian J (2005). A motor function measure for neuromuscular diseases. Construction and validation study. Neuromuscul Disord.

[14] Ricci E, Galluzzi G, Deidda G (1999). Progress in the molecular diagnosis of facioscapulohumeral muscular dystrophy and correlation between the number of KpnI repeats at the 4q35 locus and clinical phenotype. Ann Neurol.

[15] Lamperti C, Fabbri G, Vercelli L (2010). A standardized clinical evaluation of patients affected by facioscapulohumeral muscular dystrophy: The FSHD clinical score. Muscle Nerve.

[16] Santos DB, Boussaid G, Stojkovic T (2015). Respiratory muscle dysfunction in facioscapulohumeral muscular dystrophy. Neuromuscul Disord.

[17] Stubgen JP, Schultz C (2009). Lung and respiratory muscle function in facioscapulohumeral muscular dystrophy. Muscle Nerve.

[18] de Greef JC, Lemmers RJLF, Camano P (2010). Clinical features of facioscapulohumeral muscular dystrophy 2. Neurology.

[19] Chatwin M, Toussaint M, Goncalves MR (2018). Airway clearance techniques in neuromuscular disorders: a state of the art review. Respir Med.

[20] Wijkstra PJ, Meijer PM, Meinesz AF (2003). Diaphragm plication following phrenic nerve injury. Thorax.

[21] Hazenberg A, van Alfen N, Voet NB, Kerstjens HA, Wijkstra PJ (2015). Facioscapulohumeral muscular dystrophy and respiratory failure; what about the diaphragm?. Respir Med Case Rep.

